# A Branched-Chain Amino Acid-Related Metabolic Signature Characterizes Obese Adolescents with Non-Alcoholic Fatty Liver Disease

**DOI:** 10.3390/nu9070642

**Published:** 2017-06-22

**Authors:** Martina Goffredo, Nicola Santoro, Domenico Tricò, Cosimo Giannini, Ebe D’Adamo, Hongyu Zhao, Gang Peng, Xiaoqing Yu, Tukiet T. Lam, Bridget Pierpont, Sonia Caprio, Raimund I. Herzog

**Affiliations:** 1 Department of Pediatrics, Yale University School of Medicine, 330 Cedar Street, P.O. Box 208064, New Haven, CT 06520, USA; goffredo.martina@gmail.com (M.G.); cosimogiannini@hotmail.it (C.G.); ebe.dadamo@yahoo.com (E.D.); bridget.pierpont@yale.edu (B.P.); sonia.caprio@yale.edu (S.C.); 2 Department of Internal Medicine, Yale University School of Medicine, 300 Cedar Street, P.O. Box 208020, New Haven, CT 06520, USA; domenico.trico@yale.edu; 3 Department of Clinical and Experimental Medicine, University of Pisa, Pisa PI 56126, Italy; 4 Department of Pediatrics, University of Chieti, Chieti CH 66100, Italy; 5 Yale School of Public Health, New Haven, CT 06520, USA; hongyu.zhao@yale.edu (H.Z.); gang.peng@yale.edu (G.P.); xiaoqing.yu@yale.edu (X.Y.);; 6 Department of Molecular Biophysics and Biochemistry, Yale University School of Medicine, New Haven, CT 06520, USA; tukiet.lam@yale.edu

**Keywords:** metabolomics, youth, branched chain amino acids, insulin resistance, nonalcoholic fatty liver disease, obesity

## Abstract

Dysregulation of several metabolite pathways, including branched-chain amino acids (BCAAs), are associated with Non-Alcoholic Fatty Liver Disease (NAFLD) and insulin resistance in adults, while studies in youth reported conflicting results. We explored whether, independently of obesity and insulin resistance, obese adolescents with NAFLD display a metabolomic signature consistent with disturbances in amino acid and lipid metabolism. A total of 180 plasma metabolites were measured by a targeted metabolomic approach in 78 obese adolescents with (*n* = 30) or without (*n* = 48) NAFLD assessed by magnetic resonance imaging (MRI). All subjects underwent an oral glucose tolerance test and subsets of patients underwent a two-step hyperinsulinemic-euglycemic clamp and/or a second MRI after a 2.2 ± 0.8-year follow-up. Adolescents with NAFLD had higher plasma levels of valine (*p* = 0.02), isoleucine (*p* = 0.03), tryptophan (*p* = 0.02), and lysine (*p* = 0.02) after adjustment for confounding factors. Circulating BCAAs were negatively correlated with peripheral and hepatic insulin sensitivity. Furthermore, higher baseline valine levels predicted an increase in hepatic fat content (HFF) at follow-up (*p* = 0.01). These results indicate that a dysregulation of BCAA metabolism characterizes obese adolescents with NAFLD independently of obesity and insulin resistance and predict an increase in hepatic fat content over time.

## 1. Introduction

Paralleling the worldwide epidemic of childhood obesity, Non-Alcoholic Fatty Liver Disease (NAFLD) has become the most common cause of liver disease in pediatric practice, with a prevalence of ~30% in obese adolescents [1]. NAFLD encompasses a broad disease spectrum, ranging from simple steatosis to Non-Alcoholic Steatohepatitis (NASH), which in turn can progress to cirrhosis and end-stage liver disease [2]. Obese youth with NAFLD have a high risk for developing dyslipidemia, type 2 diabetes mellitus (T2DM) and cardiovascular diseases [3,4,5]. Notably, previous studies have shown that fatty liver in obese adolescents is strongly associated with hepatic and peripheral insulin resistance independent of obesity [3]. The metabolic pathways underlying these associations and in particular the development of hepatic insulin resistance remain largely unclear.

In adults, recent studies have consistently reported increased concentrations of branched-chain amino acids (BCAAs) and their metabolites in NAFLD and NASH [6,7,8,9,10,11]. Moreover, alterations in BCAAs and BCAA-derived acylcarnitines have been implicated in the development of insulin resistance and T2DM [12,13,14], thus suggesting that alterations in BCAA metabolism might play a role in the development of insulin resistance and fatty liver.

Data from adults and children are difficult to compare, since environment and growth might have an impact on metabolism. Moreover, metabolomic studies in youth have reported conflicting results [15,16,17,18,19,20,21,22,23]. A study using non-targeted metabolomic profiling in a large Hispanic cohort of children revealed different patterns of amino acids, lipids, and intermediary metabolites between obese children and their non-obese siblings [15]. Consistent with studies in adults, the plasma metabolic profile in these obese subjects was indicative of altered BCAA metabolism, insulin resistance, mitochondrial dysfunction, and reduced fatty acid beta-oxidation [15]. Other investigations in obese children have also reported similar conclusions [16,17,18,19,20]. In contrast to these results and to data from adults, a study performed in obese adolescents with and without T2DM showed no evidence of defects in fatty acid or amino acid metabolism, despite the presence of insulin resistance [22]. Another study reported a positive correlation between the disposition index (a measure of β-cell function relative to insulin sensitivity) and plasma concentrations of several metabolites, including BCAAs and BCAA-derived acylcarnitines [23]. Of note, none of these pediatric studies included measures of intrahepatic fat accumulation, a key determinant of the development of insulin resistance and youth onset of T2DM.

To fill this gap in knowledge, we applied a targeted metabolomic approach to explore whether, independently of obesity and insulin resistance per se, obese adolescents with fatty liver disease display a metabolomic signature consistent with disturbances in amino acid and lipid metabolism. Furthermore, we assessed whether metabolic alterations might predict longitudinal changes in intra-hepatic fat accumulation.

## 2. Methods

Seventy-eight adolescents (mean age of 13.3 ± 3.0 years) with a similar overall degree of obesity (similar BMI, BMI *z*-score, and total fat mass) were selected from the Yale Pediatric NAFLD Cohort from 2012 to 2015 (Table 1). Eligible subjects were children and adolescents aged 8–18 years with a BMI equal to or higher than the 95th percentile of age- and sex-specific BMI distribution. All participants had a detailed medical history and a complete physical examination including assessment of Tanner stage of development; the main exclusion criterion was presence of any liver condition besides NAFLD. To be eligible for this study, subjects could not be on medications known to affect liver function or alter glucose, amino acid, or lipid metabolism. Information relating to alcohol consumption was obtained in all subjects using our clinical lifestyle questionnaire. Autoimmune hepatitis, Wilson disease, alpha-1-antitrypsin deficiency, hepatitis B and C, and iron overload were excluded by appropriate tests in subjects with persistent elevation in alanine aminotransferase (>6 months). A standard oral glucose tolerance test (OGTT) and detailed quantification of visceral and liver fat content was performed by abdominal magnetic resonance imaging (MRI). Subjects were then divided into two groups: a high liver fat content group (HFF > 5.5%), consisting of 30 obese adolescents and a low liver fat content group (HFF < 5.5%), consisting of 48 obese adolescents. Before the formation of the two groups, we were unaware of potential differences in any of the outcomes measured.

The study was approved by the Yale University Human Investigation Committee (protocol approval number 1104008388). Written informed parental consent and written child assent were obtained from all participants. All clinical investigations have been conducted according to the principles expressed in the Declaration of Helsinki.

### 2.1. Metabolomic Profile

Fasting EDTA plasma samples were collected from study participants and were stored at −80 °C. Plasma samples were further processed according to the AbsoluteIDQ p180 (Biocrates Life Sciences AG, Innsbruck, Austria) mass spectrometry (MS)-based assay kit user manual [14]. Briefly, 10 µL of plasma were added to the filter of the upper 96-well kit plate. Twenty microliters of 5% phenyl-isothiocyanate were added to derivatize the analytes and subsequently dried again in a nitrogen evaporator. Metabolites were extracted with 300 µL of 5 mM ammonium acetate in methanol and diluted in 600 µL of running solvent (Buffer A: 0.2% formate in water). Acylcarnitines, hexoses, glycerophospholipids, and sphingolipids were quantified via flow injection analysis (FIA) and amino acids and biogenic amines by liquid chromatography/mass spectrometry (LC/MS). The LC system used was a Perkin Elmer Flexar Ultra High Pressure Liquid Chromatography System coupled in-line to a 4000 Q-Trap LC/MS system. The FIA method was carried out on the same MS system. Additionally, an Agilent Technologies ZORBAX Eclipse XDB-C18 (3.0 × 100 mm, 3.5 µm pore size) column (Santa Clara, CA, USA; part number 961967-302), coupled to an analytical Phenomenex SecurityGuard trap (C18, 4 × 3.0 mm), was utilized for the LC assay portion. Data were collected utilizing Analyst software v1.5.2 (Sciex, Framingham, MA, USA) and analyzed with Biocrates MetIDQ software v4.6.1 (Biocrates Life Sciences AG, Innsbruck, Austria). Internal standards and quality control samples of the p180 Kit were utilized to benchmark the quality of the assay, the robustness of the data, and the calculation for the concentrations of all the metabolites detected.

### 2.2. Metabolic Studies

All metabolic studies were performed at 8:00 a.m. following a 10- to 12-h overnight fast. For the purpose of this study subjects were admitted to the Yale New Haven Hospital Research Unit (HRU) of the Yale Clinical Center Investigation (YCCI).

### 2.3. Oral Glucose Tolerance Test

A standard oral glucose tolerance test (OGTT, 1.75 g glucose/kg body weight, up to 75 g glucose [24]) was conducted to determine individual glucose tolerance according to the American Diabetes Association criteria [25]. Blood samples for determination of glucose, insulin, and C-peptide were drawn at −15, 0, 30, 60, 90, 120 and 180 min. The Matsuda index was used to calculate insulin sensitivity (Whole-Body Insulin Sensitivity Index, WBISI) [26]. The insulinogenic index (IGI), which represents early phase insulin secretion and is a commonly used index of beta cell function, was calculated from the OGTT data as follows: IGI = ∆insulin (0–30 min) in microunits per milliliter divided by the glucose (0–30 min) in milligrams per deciliter. The disposition index (DI), which provides an integrate picture of glucose tolerance including both insulin sensitivity and insulin secretion, was calculated as the product of the IGI and the WBISI, based on the curvilinear relation of these OGTT derived parameters previously described by our group in obese children and adolescents [27].

### 2.4. Hyperinsulinemic-Euglycemic Clamp

A subset of 12 subjects agreed to undergo a 2-step hyperinsulinemic-euglycemic clamp. After local infiltration with lidocaine, two intravenous catheters were inserted in the antecubital vein of each arm, one for blood sampling and one for co-infusion of 6,6-^2^D-glucose to further assess muscle, hepatic, and peripheral insulin sensitivity, as previously reported [28]. The sampling arm was kept in a heated box for arterialization of blood. Whole-body insulin sensitivity was measured via a two-step euglycemic clamp by infusing insulin as a primed continuous infusion at 4 and 80 mU/m^2^·min. Each step lasted 2 h. A primed continuous infusion of 6,6-^2^D-glucose was used to quantify the effects of insulin on glucose turnover. To maintain the plasma enrichment of 6,6-^2^D-glucose constant at baseline value throughout the clamp, the “Hot GINF” method was used, as previously reported [29]. Arterialized blood samples were collected every 10 min during the last 30 min of the baseline period and during each step of the clamp for measurement of glucose enrichments, hormones, and substrates.

### 2.5. Abdominal Magnetic Resonance Imaging

Liver fat content was measured by MRI on a GE or Siemens Sonata 1.5 Tesla system [30], using the two-point Dixon (2PD) method as modified by Fishbein and colleagues [31]. Briefly, using the MRIcro software program (Dr. Chris Rorden, University of South Carolina, Columbia, SC, USA; available at http://people.cas.sc.edu/rorden/mricro/mricro.html), five regions of interest were drawn on each image and the mean pixel signal intensity level was recorded. HFF was calculated in duplicate from the mean pixel signal intensity data using the formula: [(Sin-Sout)/(2 × Sin)] × 100. The imaging parameters were: matrix size = 128 × 256, flip angle (α) = 30°, TR = 18 ms, TEs = 2.38/4.76 ms out-of-phase and in-phase, respectively, bandwidth = 420 Hz/pixel, six averages, slice thickness = 10 mm, one slice, 2.3 s/slice (for 2 points), scan time = 14 s in a single breath-hold [31].

A subset of 23 children gave their consent to participate in longitudinal follow up and to undergo a second MRI after ~2 years. The rationale for the 2-year time interval was to evaluate changes in HFF over a relatively short period, while minimizing bias introduced by environmental factors.

### 2.6. Statistical Analyses

All variables were tested for normal distribution and appropriately log transformed when required. Of 180 metabolites measured, 12 with more than 25% missing data were removed, leaving 168 metabolites for further analysis. The median of each metabolite data set was used as imputed value for the remaining missing data. A Random Forest method was employed. Random Forest is a supervised classification method that builds hundreds of decision trees from subsets of original data and then combines the decision trees together as a forest to vote for the final result [32,33]. Only some of the samples are used to construct each decision tree. This provides an unbiased estimation of classification error with each case without cross-validation omitted. For a classification of two categories, the importance of each variable can be measured by mean decrease of the variability or “Gini” index. If a variable is of importance, its absence during the permutations of decision tree construction results in a big drop in the Gini index. Otherwise, the influence would be small. We conducted two kinds of Random Forest analyses. The first included all metabolites while the second iteration included only the top 12 most relevant metabolites with big mean decreases of Gini index during the first analysis. Differences in plasma concentrations of all 168 metabolites between subjects with and without NAFLD were evaluated by Student’s *t*-tests. Differences between groups were also evaluated by a general linear model (GLM) adjusting for age, gender, ethnicity, BMI, and whole-body insulin sensitivity index (WBISI). A multiple testing correction via false discovery rate (FDR) according to the Group Benjamini–Hochberg procedure [34] was used as appropriate. A Spearman correlation was performed to evaluate the association between BCAA concentration and the suppression of hepatic glucose production, glycerol turnover, and glucose disposal rate. In the longitudinal study, paired Student’s *t*-tests were used to analyze changes between baseline and follow-up characteristics of the subjects enrolled and differences in baseline metabolite levels between “progressors” (deltaHFF > 0) and “non-progressors” (deltaHFF ≤ 0). The performance of baseline valine levels to predict HFF progression as a categorical variable was assessed by the c statistic (the area under the receiver operating characteristic curve (ROC AUC)). Statistical tests were conducted using a two-sided α- level of 0.05. Data are represented as mean ± standard deviation. Statistical analyses were performed using SAS 9.4 (SAS Institute Inc., Cary, NC, USA).

## 3. Results

### 3.1. Anthropometric and Metabolic Profiles

To dissociate the confounding effects of obesity from that of fatty liver on the metabolomic profile, we recruited adolescents with a similar overall degree of obesity with or without NAFLD. Thus, the two groups of subjects were matched for BMI, BMI *z*-score, and body fat, as well as for age (Table 1). However, the two groups differed for gender and ethnicity, reflecting the higher prevalence of NAFLD in boys and in Hispanics described in literature [35]. As previously reported, obese adolescents with fatty liver, independent of obesity, had elevated levels of insulin, triglycerides, and transaminases, along with the presence of reduced whole-body insulin sensitivity (WBISI) [36]. Clinical and metabolic characteristics of the two subgroups of subjects who underwent a hyperinsulinemic-euglycemic clamp and/or a follow-up abdominal MRI are shown in Tables S1 and S2, respectively, and were not different from those of the whole study population (Table S3).

### 3.2. Metabolomics Signature of Obese Youth with NAFLD

**Figure 1:**
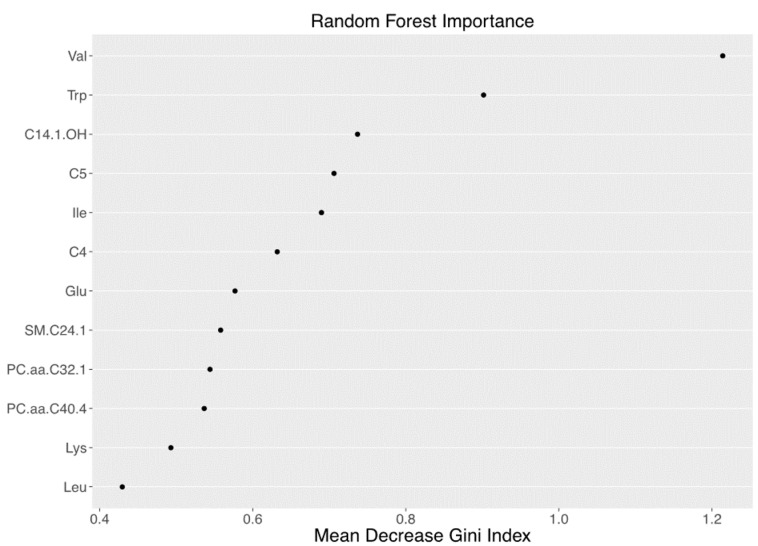
Random forest importance plot for subjects without vs. with Non-Alcoholic Fatty Liver Disease (NAFLD). Random forest plot of top-ranked metabolites with the highest Mean Decreased Gini index for the classification of obese adolescents with and without NAFLD.

**Figure 2:**
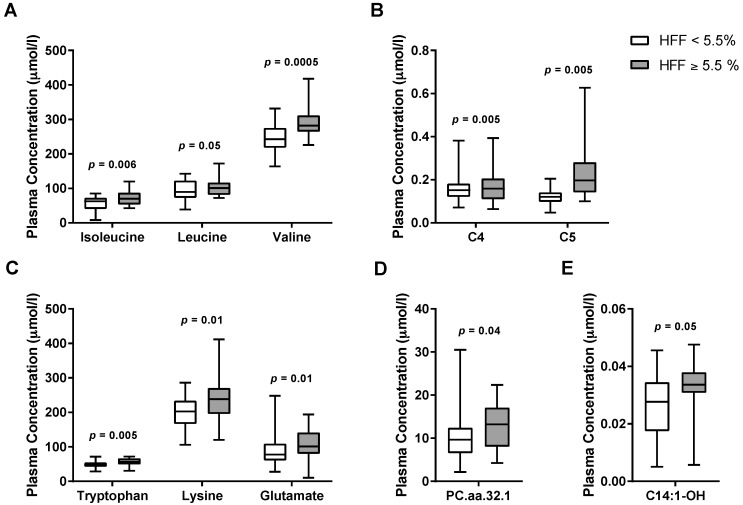
Metabolites associated with NAFLD in obese youth: (**A**) BCAAs, branched-chain amino acids (isoleucine, leucine, and valine); (**B**) C4 and C5; (**C**) tryptophan, lysine, and glutamate; (**D**) PC.aa.C32.1 (long-chain phosphatidylcholine C32:1); and (**E**) C14:1-OH (Hydroxytetradecenoylcarnitine). Statistical comparisons between the two groups were made by Student’s *t*-tests.

Random Forest (RF) analysis revealed that the top 12 metabolites had a sensitivity of 68.9% and specificity of 64.7% to detect NAFLD (Figure 1). Of the original 168 compounds (Table S4, Figures S1–S4), 10 metabolites showed a difference between the two groups in the preliminary analysis; in particular, subjects with NAFLD showed higher plasma levels of BCAAs (valine, leucine, isoleucine), tryptophan, lysine, glutamate, as well as C4, C5, and C14:1-OH carnitine esters, and long-chain phosphatidylcholine C32:1 (PC.aa.C32.1) (Figure 2). After correction for multiple comparisons and adjustment for confounding factors (age, gender, ethnicity, BMI, and insulin sensitivity), differences in plasma valine (*p* = 0.02), isoleucine (*p* = 0.03), tryptophan (*p* = 0.02), and lysine (*p* = 0.02) remained statistically significant.

### 3.3. Associations between Branched-Chain Amino Acids and Hepatic Insulin Sensitivity

**Figure 3:**
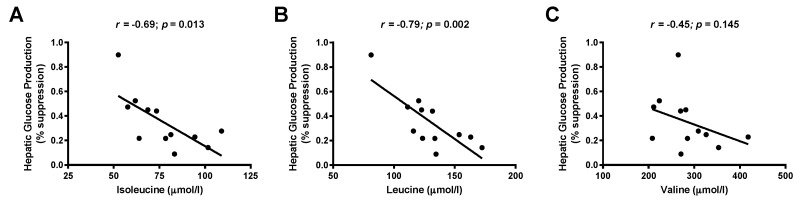
Association between hepatic glucose suppression during a two-step hyperinsulinemic-euglycemic clamp and: (**A**) isoleucine; (**B**) leucine; and (**C**) valine concentrations. Rank correlations between variables are indicated by Spearman’s rank correlation coefficient (*r*).

We found a negative correlation between the OGTT-derived WBISI and plasma isoleucine (*r* = −0.318; *p* = 0.005) and valine (*r* = −0.369; *p* = 0.001) levels in the whole study population. Moreover, the same amino acids were even more strongly associated with glucose disposal rate, a marker of muscle insulin resistance derived from hyperinsulinemic clamp studies (isoleucine *r* = −0.61, *p* = 0.036; valine *r* = −0.58, *p* = 0.048). We also observed an independent association between BCAAs and the percent suppression of hepatic glucose production during the first step (low insulin dose) of the clamp (*r* = −0.70; *p* = 0.013 for isoleucine and *r* = −0.79; *p* = 0.002 for leucine), as shown in Figure 3. Overall, these data show that circulating BCAAs were associated with peripheral and hepatic insulin sensitivity.

### 3.4. Correlation between Metabolite Concentration and Changes of Hepatic Fat Content over Time

**Figure 4:**
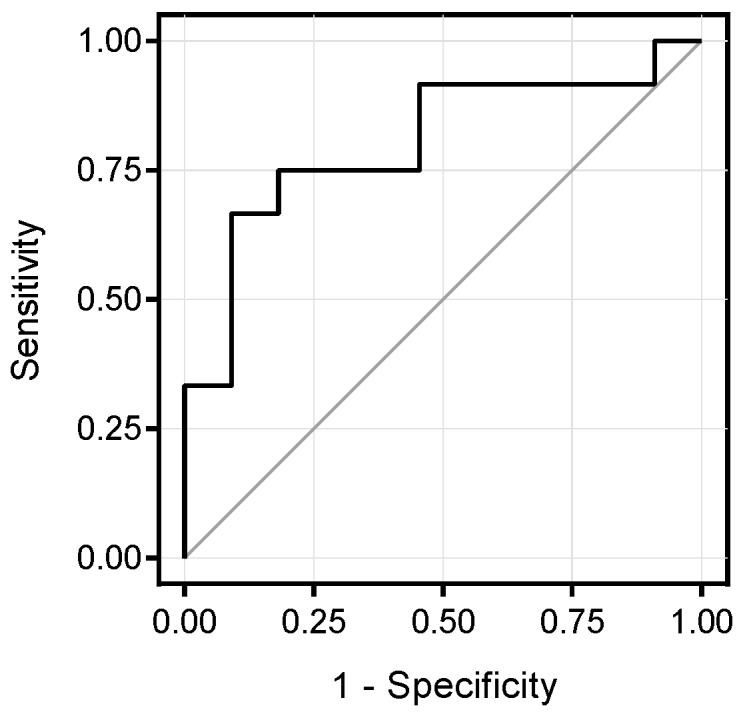
Receiver operating characteristic (ROC) curve of valine for predicting HFF progression in obese youth. The area under the ROC curve was 0.803. With a cutoff of 276 μmol, valine levels had 66.7% sensitivity and 90.9% specificity to predict increases in HFF.

Hepatic fat content (HFF) from 23 children was re-evaluated after a follow up of 2.2 ± 0.8 years (Table S2). After the follow-up, there was a 34% increase in HFF (*p* = 0.02) and an 8% increase in BMI (*p* = 0.0003). Interestingly, higher baseline levels of valine were identified in children whose HFF increased during the follow-up period (*n* = 12; 296 ± 53 μmol/L) than in those subjects whose HFF remained stable or decreased (*n* = 11; 246 ± 29 μmol/L; *p* = 0.01). The area under the ROC curve of plasma valine for predicting HFF progression in obese youth was 0.803 (Figure 4). Valine levels higher than 276 μmol had 66.7% sensitivity and 90.9% specificity to predict increases in HFF.

## 4. Discussion

In this study, we observed that obese adolescents with fatty liver disease show a metabolomic signature characterized by elevated plasma amino acids including two BCAAs (valine and isoleucine), tryptophan, and lysine that is independent of obesity and insulin resistance. Importantly, we found that BCAA levels are associated with the degree of hepatic and peripheral insulin resistance; in particular, high concentrations of BCAAs were negatively correlated to the ability of insulin to suppress hepatic glucose production as well as to the glucose disposal rate. Moreover, baseline valine levels were predictive of longitudinal changes in liver fat accumulation during the two-year follow-up. These findings are of particular relevance as they suggest that an early alteration in the metabolism of BCAAs might play a role in the development and progression of NAFLD among obese youth.

In accordance with these data, observational and metabolomic studies have consistently shown an increase of circulating amino acid levels, particularly BCAAs, in adults with insulin-resistant obesity, T2DM, and NAFLD/NASH [6,7,8,9,10,11,12,13,14,37,38,39]. Furthermore, increased plasma concentrations of BCAAs and BCAA-byproducts in obese children were reported in most pediatric studies [15,17,18,19,40], including investigations in two large cohorts of 803 [15] and 262 [17] children. In these studies, high BCAA levels showed a clear association with adiposity and markers of insulin resistance [15,16,17,18,19,40]. Moreover, an alteration of BCAA metabolism was recently found in a small cohort of obese adolescents with NAFLD (*n* = 9) [40]. However, a metabolomic study from Wahl et al. [20] did not observe increased BCAA levels in obese children, and two studies reported even lower BCAA concentrations in obese adolescents with or without dysglycemia compared with lean subjects [22,23]. These discrepancies may be attributed to differences in subject characteristics (age, gender, ethnicity, and glycemic control), analytic platforms, and presence or absence of fatty liver disease. Indeed, none of these previous pediatric studies has combined measures of intrahepatic fat accumulation and state-of-the-art measurement of insulin resistance via hyperinsulinemic-euglycemic clamps. In patients with fatty liver disease, a significant down-regulation of BCAA catabolism in adipose tissue, but not in liver or skeletal muscle, has been shown [10,11,41]. This was attributed to reduced mitochondrial activity [11] or to the activation of inflammatory pathways in the adipose tissue [41,42]. Notably, the rate of BCAA metabolism was inversely correlated with plasma BCAA concentrations, liver fat content, and indexes of insulin resistance [11].

Interestingly, we showed that other amino acids, such as tryptophan, lysine, and glutamate, are elevated in obese subjects with NAFLD. It is conceivable that they may compete with BCAAs for amino acid transporters on target organs, resulting in a reduced BCAA tissue uptake and catabolism [43,44,45]. In addition, changes in gut microbiota, which have recently been proposed as key contributors to the pathogenesis of NAFLD [46], may raise circulating BCAAs [47,48], by stimulating de novo amino acid biosynthesis [48,49], by slowing BCAA catabolism [48], or by increasing gut permeability [47,50].

Longitudinal studies in adults [51] and children [19] have previously reported that increased plasma BCAA concentrations independently predict development and progression of insulin resistance or T2DM. In the present study, we show that valine levels are predictive of longitudinal changes in liver fat accumulation in obese children. Although it is reasonable to speculate that early impairments of BCAA metabolism might contribute to the development and progression of NAFLD and insulin resistance in obese subjects, whether elevated concentrations of BCAAs and their metabolites are causative factors or only biomarkers remains unclear and will require further validation in dedicated prospective studies. Several pathways linking high concentrations of BCAAs and BCAA byproducts to intra-hepatic fat accumulation in NAFLD have been hypothesized [52].

Elevated BCAAs might lead to hepatic insulin resistance either directly by activating gluconeogenesis [52] or indirectly through persistent activation of mTOR complex 1 (mTORC1), which promotes the inhibitory serine phosphorylation of insulin receptor substrate 1 (IRS-1) [38]. Increased BCAAs may also lead to peripheral insulin resistance by promoting accumulation of incompletely oxidized lipids in skeletal muscle. The underlying mechanisms include an increased substrate load, which decrease the efficiency of fatty acid oxidation [53], and the activation of endothelial fatty acid transporters through the paracrine action of valine metabolite 3-hydroxybutyrate (3-HIB) [13]. Increased hepatic and peripheral insulin resistance result in glucose excess that in turn might sustain hepatic de novo lipogenesis and fat accumulation [52]. On the other hand, insulin resistance can also cause both a reduction of BCAA catabolism and an increase of BCAA rate of appearance, which might perpetuate BCAA plasma level elevation in the blood [38]. Taken together, these considerations clearly indicate the presence of a vicious circle linking high BCAA concentrations, insulin resistance, and NAFLD, so that it is difficult to define the ultimate cause of this complex metabolic pattern. From a clinical point of view, these observations may support the hypothesis that pharmacologic manipulation of the pathways involved in BCAA metabolism might yield benefits like improvement in hepatic and whole-body insulin sensitivity and a reduction of liver fat content in obese subjects.

The strengths of the present study include the enrollment of a well-characterized group of adolescents carefully matched for the level of overall obesity, as to minimize the known effects of obesity on amino acid and lipid metabolism. Plasma samples were obtained at rest following an overnight fast and analyzed with a metabolomic approach using Random Forest analysis. By using a targeted metabolomic approach, we quantified precisely and accurately 180 endogenous metabolites from five different compound classes (i.e., acylcarnitines, amino acids, hexoses, phospho- and sphingolipids, and biogenic amines), thereby covering key metabolic pathways. Age, gender, ethnicity, BMI, insulin sensitivity, and visceral fat were accounted for in statistical analyses as possible confounding factors. Assessment of liver steatosis was performed in all subjects by using a validated, quantitative, non-invasive MRI method [31], and was repeated in a subgroup of subjects to measure longitudinal changes in hepatic fat accumulation. However, some limitations should also be considered. In comparison to an untargeted metabolomics approach, targeted metabolomics does not allow a comprehensive analysis of all measurable analytes in plasma samples, particularly of chemical unknowns. Due to the observational design of the study, we could not establish causality and address how BCAAs can affect hepatic fat content and changes in insulin resistance, something that will require dedicated prospective studies in the future. The number of subjects who underwent a hyperinsulinemic-euglycemic clamp was relatively small; however, the demonstrated associations between BCAAs and indexes of insulin sensitivity were clear-cut and further confirmed in the whole population by the OGTT-derived WBISI. In addition, conclusions based on failure to reject the null hypothesis have been avoided.

## 5. Conclusions

In summary, high plasma concentrations of BCAAs are associated with intra-hepatic fat content, independently of the degree of obesity and insulin resistance, and may represent the link between hepatic insulin resistance and NAFLD in obese youth. Understanding the signaling and metabolic pathways of BCAAs could point towards novel potential therapeutic targets for the treatment of NAFLD.

## Figures and Tables

**Table 1:** Characteristics of the study population stratified by hepatic fat content (HFF %).

	HFF < 5.5% (*n* = 48)	HFF ≥ 5.5% (*n* = 30)	*p*
**CLINICAL FEATURES**			
Age (years)	13.6 ± 3.0	12.8 ± 2.8	0.30
Gender (M/F)	17/31 (35%/65%)	21/9 (70%/30%)	0.003
Race (Caucasian/African American/Hispanic/Asian)	15/19/13/1 (31%/40%/27%/2%)	11/3/16/0 (37%/10%/53%/0%)	0.02
Glucose tolerance (NGT/IGT)	41/7 (85%/15%)	20/10 (67%/33%)	0.10
BMI (kg/m^2^)	34.1 ± 7.2	33.6 ± 5.1	0.64
BMI *z*-score	2.2 ± 0.3	2.3 ± 0.2	0.24
Body Fat (%)	44.27 ± 7.17	45.35 ± 7.53	0.34
**GLUCOSE METABOLISM**			
Fasting glucose (mg/dL)	91 ± 7	94 ± 6	0.15
Fasting insulin (µU/mL)	25 ± 11.3	45.4 ± 23.9	<0.001
2 h glucose (mg/dL)	114 ± 22	128 ± 28	0.01
Hemoglobin A_1C_ (%)	5.4 ± 0.2	5.5 ± 0.3	0.31
WBISI	2.12 ± 0.89	1.2 ± 0.8	<0.001
IGI	4.1 ± 2.8	5.93 ± 5.6	0.08
DI	8.0 ± 6.4	6.7 ± 7.1	0.50
**LIPID PROFILE**			
Total Cholesterol (mg/dL)	148 ± 26	159 ± 36	0.19
HDL Cholesterol (mg/dL)	45 ± 9	42.47 ± 10	0.23
LDL Cholesterol (mg/dL)	87 ± 23	92 ± 30	0.55
Triglycerides (mg/dL)	78 ± 38	127 ± 80	0.002
**LIVER FUNCTION**			
Alanine Transaminase (U/L)	16.5 ± 6.9	40.2 ± 28.5	<0.001
Aspartate Transaminase (U/L)	19.7 ± 4.3	30.3 ± 15.0	0.001
**BODY FAT COMPOSITION**			
Visceral (cm^2^)	55.6 ± 28.6	79.7 ± 24.8	<0.001
Deep Subcutaneous (cm^2^)	188.6 ± 141.6	177.1 ± 57.1	0.40
Subcutaneous (cm^2^)	552.1 ± 224.5	527.9 ± 167.4	0.90
Superficial Subcutaneous (cm^2^)	168.1 ± 70.3	129.8 ± 52.8	0.002
Deep/Superficial Subcutaneous	1.1 ± 0.4	1.5 ± 0.5	0.02
Hepatic Fat Fraction (%)	1.1 ± 1.6	18.3± 10.2	<0.001

HFF, hepatic fat content; BMI, Body Mass Index; DI, Disposition Index; IGI, Insulinogenic index; NGT, Normal Glucose tolerance; IGT, Impaired Glucose Tolerance; WBISI, Whole Body Insulin Sensitivity Index. Statistical comparisons between the two groups were made by either Student’s *t*-tests for continuous variables or Chi-square tests for categorical variables.

## Supplementary Materials

The following are available online at www.mdpi.com/2072-6643/9/7/642/s1, Figure S1: Heatmap of the analyzed metabolites, Figure S2: Correlation between metabolites, Figure S3: Percentage of variation for the first 9 principal components, Figure S4: Score plot for the first 9 principal components, Table S1: Characteristics of subjects who underwent a 2-step hyperinsulinemic-euglycemic clamp stratified by hepatic fat content (HFF), Table S2: Characteristics of subjects included in the longitudinal study at baseline and after a follow-up of 2.2 ± 0.8 years, Table S3: Comparisons between the characteristics of the whole study population (*n* = 78) and the two subgroups of subjects who underwent the hyperinsulinemic-euglycemic clamp or the follow-up examination, Table S4: List and abbreviations of the analyzed metabolites.
