# Supplementary material for: A Branched-Chain Amino Acid-Related Metabolic Signature Characterizes Obese Adolescents with Non-Alcoholic Fatty Liver Disease
Source: Nutrients. 2017 Jun 22;9(7):642. doi: 10.3390/nu9070642 (PMC5537762; doi:10.3390/nu9070642)

Table S1. Characteristics of subjects who underwent a 2-step hyperinsulinemic-euglycemic clamp stratified by hepatic fat content (HFF).

	**HFF < 5.5%** (*n*=7)	**HFF ≥ 5.5%** (*n*=5)	***p***
**CLINICAL FEATURES**			
Age (years)	15.3 ± 2.7	13. 9 ± 2.6	**0.40**
Gender (M/F)	1/6 (14%/ 86%)	3/2 (60%/ 40%)	**0.15**
Race (Caucasian/African American/Hispanic/Asian)	3/4/0/0 (43% /75% /0% /0%)	1/0/4/0 (20% /0% /80% /0%)	**0.01**
Glucose tolerance (NGT/IGT)	6/1 (86%/ 14%)	4/1 (80%/ 20%)	**0.68**
BMI (kg/m^2)	36.5 ± 5.4	35.1 ± 5.3	**0.67**
BMI *z*-score	2.3 ± 0.2	2.4 ± 0.1	**0.22**
Body Fat (%)	46.9 ± 4	45.9 ± 4.8	**0.66**
**GLUCOSE METABOLISM**			
Fasting glucose (mg/dl)	95 ± 6	93 ± 7.6	**0.62**
Fasting insulin (µU/ml)	29.6 ± 12.4	26.7 ± 7.6	**0.91**
2 h glucose (mg/dl)	116.7 ± 19.3	122 ± 26.2	**0.69**
Hemoglobin A_{1C} (%)	5.4 ± 0.33	5.5 ± 0.3	**0.42**
WBISI	2 ± 1.1	1.9 ± 1.1	**0.91**
IGI	4.8 ± 3.1	3.2 ± 0.8	**0.31**
DI	8.2 ± 4.8	5.6 ± 2.1	**0.28**
**LIPID PROFILE**			
Total Cholesterol (mg/dL)	158.2 ± 23	194 ± 62	**0.21**
HDL Cholesterol (mg/dL)	42.7 ± 5.6	47.2 ± 5.3	**0.20**
LDL Cholesterol (mg/dL)	93.7 ± 17.3	121.6 ± 50.1	**0.23**
Triglycerides (mg/dL)	109.5 ± 59.6	126.4 ± 85.2	**0.71**
**LIVER FUNCTION**			
Alanine Transaminase (U/L)	13.3 ± 4.1	21 ± 11.5	**0.16**
Aspartate Transaminase (U/L)	17.8 ± 2.6	20.4 ± 3.1	**0.17**
**BODY FAT COMPOSITION**			
Visceral (cm^2)	74.7 ± 36.3	83.4 ± 41.5	**0.71**
Deep Subcutaneous (cm^2)	223.7 ± 83	209.9 ± 72.5	**0.77**
Subcutaneous (cm^2)	631.7 ± 177	574 ± 148.7	**0.57**
Superficial Subcutaneous (cm^2)	165.3 ± 49.2	121.6 ± 41.3	**0.15**
Deep/Superficial Subcutaneous	1.3 ± 0.2	1.7 ± 0.4	**0.05**
Hepatic Fat Fraction (%)	1.6 ± 2.1	13 ± 7.4	**0.003**

BMI, Body Mass Index; DI, Disposition Index; IGI, Insulinogenic index; NGT, Normal Glucose tolerance; IGT, Impaired Glucose Tolerance; WBISI, Whole Body Insulin Sensitivity Index. Statistical comparisons between the two groups were made by either Student`s *t*-tests for continuous variables or Chi-square tests for categorical variables.

Table S2. Characteristics of subjects included in the longitudinal study at baseline and after a follow-up of 2.2±0.8 years.

	**Baseline** (*n*=23)	**Follow-up** (*n*=23)	***p***
**CLINICAL FEATURES**			
Age (years)	13.9 ± 2.8	16.3 ± 2.9	**<0.0001**
Gender (M/F)	9/14 (40%/ 60%)	-	**-**
Race (Caucasian/African American/Hispanic/Asian)	10/7/6/0 (44%/ 30%/ 26%/ 0%)	-	**-**
Glucose tolerance (NGT/IGT)	21/2 (91%/ 9%)	22/1 (95%/ 5%)	**0.55**
BMI (kg/m^2)	35.4 ± 6.6	38.3 ± 6.3	**0.0003**
BMI *z*-score	2.3 ± 0.3	2.3 ± 0.3	**0.68**
Body Fat (%)	43.7 ± 7.1	44.0 ± 6.0	**0.67**
**GLUCOSE METABOLISM**			
Fasting glucose (mg/dl)	93.1 ± 6.5	91.1 ± 8.0	**0.27**
Fasting insulin (µU/ml)	34.7 ± 20.5	37.2 ± 22.8	**0.49**
2 h glucose (mg/dl)	110 ± 20	109 ± 18	**0.72**
Hemoglobin A_{1C} (%)	5.5 ± 0.3	5.5 ± 0.2	**0.73**
WBISI	2.0 ± 1.1	1.8 ± 0.9	**0.31**
IGI	5.6 ± 6.3	5.1 ± 3.0	**0.61**
DI	8.8 ± 7.9	7.7 ± 4.2	**0.39**
**LIPID PROFILE**			
Total Cholesterol (mg/dL)	156.0 ± 26.8	145.3 ± 16.2	**0.14**
HDL Cholesterol (mg/dL)	44.7 ± 9.5	43.9 ± 12.9	**0.84**
LDL Cholesterol (mg/dL)	93.5 ± 22.5	83.2 ±13.1	**0.09**
Triglycerides (mg/dL)	88.9 ± 47.3	86.8 ± 52.3	**0.87**
**LIVER FUNCTION**			
Alanine Transaminase (U/L)	22.1 ± 19.7	23.5 ± 17.3	**0.36**
Aspartate Transaminase (U/L)	23.0 ± 9.3	23.5 ± 10.7	**0.85**
**BODY FAT COMPOSITION**			
Visceral (cm^2)	63.4 ± 28.5	69.1 ± 36.1	**0.28**
Deep Subcutaneous (cm^2)	197 ± 77	195 ± 67	**0.86**
Subcutaneous (cm^2)	565 ± 175	553 ± 203	**0.77**
Superficial Subcutaneous (cm^2)	158 ± 56	175 ± 65	**0.68**
Deep/Superficial Subcutaneous	1.3 ± 0.3	1.7 ± 0.5	**0.17**
Hepatic Fat Fraction (%)	5.3 ± 10.0	7.1 ± 11.5	**0.02**

BMI, Body Mass Index; DI, Disposition Index; IGI, Insulinogenic index; NGT, Normal Glucose tolerance; IGT, Impaired Glucose Tolerance; WBISI, Whole Body Insulin Sensitivity Index. Statistical comparisons between the two groups were made by either Student`s *t*-tests for continuous variables or Chi-square tests for categorical variables.

Table S3. Comparisons between the characteristics of the whole study population (*n*=78) and the two subgroups of subjects who underwent the hyperinsulinemic-euglycemic clamp or the follow-up examination.

	**Clamp group All** (*n*=23) *p*	**Clamp group HFF < 5.5%** (*n*=17) *p*	**Clamp group HFF ≥ 5.5%** (*n*=6) *p*	**Follow-up group All** (*n*=12) *p*
**CLINICAL FEATURES**				
Age (years)	*0.23*	*0.43*	*0.47*	*0.16*
Gender (M/F)	*0.48*	*0.87*	*0.77*	*0.37*
Race (Caucasian/African American/Hispanic/Asian)	*0.51*	*0.66*	*0.61*	*0.98*
Glucose tolerance (NGT/IGT)	*0.41*	*0.42*	*0.77*	*0.88*
BMI (kg/m^2)	*0.34*	*0.83*	*0.35*	*0.27*
BMI *z*-score	*0.79*	*0.87*	*0.59*	*0.99*
Body Fat (%)	*0.73*	*0.25*	*0.86*	*0.35*
**GLUCOSE METABOLISM**				
Fasting glucose (mg/dl)	*0.37*	*0.50*	*0.29*	*0.42*
Fasting insulin (μU/ml)	*0.90*	*0.55*	*0.71*	*0.78*
2 h glucose (mg/dl)	*0.21*	*0.06*	*0.95*	*0.77*
Hemoglobin A_{1C} (%)	*0.70*	*0.84*	*0.64*	*0.98*
WBISI	*0.59*	*0.64*	*0.83*	*0.77*
IGI	*0.84*	*0.98*	*0.66*	*0.56*
DI	*0.33*	*0.52*	*0.64*	*0.79*
**LIPID PROFILE**				
Total Cholesterol (mg/dL)	*0.60*	*0.47*	*0.75*	*0.18*
HDL Cholesterol (mg/dL)	*0.92*	*0.20*	*0.31*	*0.84*
LDL Cholesterol (mg/dL)	*0.52*	*0.42*	*0.75*	*0.24*
Triglycerides (mg/dL)	*0.64*	*0.12*	*0.43*	*0.38*
**LIVER FUNCTION**				
Alanine Transaminase (U/L)	*0.17*	*0.90*	*0.40*	*0.10*
Aspartate Transaminase (U/L)	*0.57*	*0.47*	*0.62*	*0.12*
**BODY FAT COMPOSITION**				
Visceral (cm^2)	*0.89*	*0.67*	*0.57*	*0.29*
Deep Subcutaneous (cm^2)	*0.23*	*0.35*	*0.35*	*0.08*
Subcutaneous (cm^2)	*0.45*	*0.55*	*0.64*	*0.20*
Superficial Subcutaneous (cm^2)	*0.61*	*0.97*	*0.95*	*0.88*
Deep/Superficial Subcutaneous	*0.54*	*0.37*	*0.43*	*0.054*
Hepatic Fat Fraction (%)	*0.26*	*0.61*	*0.83*	*0.76*

BMI, Body Mass Index; DI, Disposition Index; IGI, Insulinogenic index; NGT, Normal Glucose tolerance; IGT, Impaired Glucose Tolerance; WBISI, Whole Body Insulin Sensitivity Index. Statistical comparisons between groups were made by either Student`s t-tests or Wilcoxon test for continuous variables and by Chi-square test for categorical variables as appropriate.

Table S4. List and abbreviations of the analyzed metabolites.

Abbreviation	Full biochemical name
**AMINO ACIDS**	
Ala	Alanine
Arg	Arginine
Asn	Asparagine
Asp	Aspartic acid
Cys	Cysteine
Gln	Glutamine
Glu	Glutamic acid
Gly	Glycine
His	Histidine
Ile	Isoleucine
Leu	Leucine
Lys	Lysine
Met	Methionine
Orn	Ornithine
Phe	Phenylanine
Pro	Proline
Ser	Serine
Thr	Threonine
Trp	Tryptophan
Tyr	Tyrosine
Val	Valine
Met-SO	Methionine Sulfoxide
total DMA	Total Dimethylamine
**ACYLCARNITINES**	
C0	DL-Carnitine
C10	Decanoyl-L-carnitine
C10:1	Decenoyl-L-carnitine
C10:2	Decadienyl-L-carnitine
C12	Dodecanoyl-L-carnitine
C12-DC	Dodecanedioyl-L-carnitine
C12:1	Dodecenoyl-L-carnitine
C14	T etradecanoyl-L-carnitine
C14:1	T etradecenoyl-L-carnitine
C14:1-OH	Hydroxytetradecenoyl-L-carnitine
C14:2	T etradecadienyl-L-carnitine
C14:2-OH	Hydroxytetradecadienyl-L-carnitine
C16	Hexadecanoyl-L-carnitine
C16-OH	Hydroxyhexadecanoyl-L-carnitine
C16:1	Hexadecenoyl-L-carnitine
C16:1-OH	Hydroxyhexadecenoyl-L-carnitine
C16:2	Hexadecadienyl-L-carnitine
C16:2-OH	Hydroxyhexadecadienyl-L-carnitine
C18	Octadecanoyl-L-carnitine

C18:1	Octadecenoyl-L-carnitine
C18:1-OH	Hydroxyoctadecenoyl-L-carnitine
C18:2	Octadecadienyl-L-carnitine
C2	Acetyl-L-carnitine
C3	Propionyl-L-carnitine
C3-DC (C4-OH)	Malonyl-L-carnitine / Hydroxybutyryl-L-carnitine
C3-OH	Hydroxypropionyl-L-carnitine
C3:1	Propenyl-L-carnitine
C4	Butyryl-L-carnitine
C4:1	Butenyl-L-carnitine
C5	Valeryl-L-carnitine
C5-DC (C6-OH)	Glutaryl-L-carnitine / Hydroxyhexanoyl-L-carnitine
C5-M-DC	Methylglutaryl-L-carnitine
C5-OH (C3-DC-M)	Methylmalonyl-L-carnitine / Hydroxyvaleryl-L-carnitine
C5:1	Tiglyl-L-carnitine
C5:1-DC	Glutaconyl-L-carnitine
C6 (C4:1-DC)	Glutaryl-L-carnitine / Hydroxyhexanoyl-L-carnitine
C6:1	Hexenoyl-L-carnitine
C7-DC	Pimelyl-L-carnitine
C8	Octanoyl-L-carnitine
C9	Nonayl-L-carnitine
**LYSOPHOSPHATIDYLCHOLINES**	
lysoPC a C14:0	Lysophosphatidylcholine acyl C14:0
lysoPC a C16:0	Lysophosphatidylcholine acyl C16:0
lysoPC a C16:1	Lysophosphatidylcholine acyl C16:1
lysoPC a C17:0	Lysophosphatidylcholine acyl C17:0
lysoPC a C18:0	Lysophosphatidylcholine acyl C18:0
lysoPC a C18:1	Lysophosphatidylcholine acyl C18:1
lysoPC a C18:2	Lysophosphatidylcholine acyl C18:2
lysoPC a C20:3	Lysophosphatidylcholine acyl C20:3
lysoPC a C20:4	Lysophosphatidylcholine acyl C20:4
lysoPC a C24:0	Lysophosphatidylcholine acyl C24:0
lysoPC a C26:0	Lysophosphatidylcholine acyl C26:0
lysoPC a C26:1	Lysophosphatidylcholine acyl C26:1
lysoPC a C28:0	Lysophosphatidylcholine acyl C28:0
lysoPC a C28:1	Lysophosphatidylcholine acyl C28:1
**DIACYL PHOSPHATIDYLCHOLINES**	
PC aa C24:0	Phosphatidylcholine diacyl C24:0
PC aa C26:0	Phosphatidylcholine diacyl C26:0
PC aa C28:1	Phosphatidylcholine diacyl C28:1
PC aa C30:0	Phosphatidylcholine diacyl C30:0
PC aa C30:2	Phosphatidylcholine diacyl C30:2
PC aa C32:0	Phosphatidylcholine diacyl C32:0
PC aa C32:1	Phosphatidylcholine diacyl C32:1
PC aa C32:2	Phosphatidylcholine diacyl C32:2
PC aa C32:3	Phosphatidylcholine diacyl C32:3
PC aa C34:1	Phosphatidylcholine diacyl C34:1

PC aa C34:2	Phosphatidylcholine diacyl C34:2
PC aa C34:3	Phosphatidylcholine diacyl C34:3
PC aa C34:4	Phosphatidylcholine diacyl C34:4
PC aa C36:0	Phosphatidylcholine diacyl C36:0
PC aa C36:1	Phosphatidylcholine diacyl C36:1
PC aa C36:2	Phosphatidylcholine diacyl C36:2
PC aa C36:3	Phosphatidylcholine diacyl C36:3
PC aa C36:4	Phosphatidylcholine diacyl C36:4
PC aa C36:5	Phosphatidylcholine diacyl C36:5
PC aa C36:6	Phosphatidylcholine diacyl C36:6
PC aa C38:0	Phosphatidylcholine diacyl C38:0
PC aa C38:1	Phosphatidylcholine diacyl C38:1
PC aa C38:3	Phosphatidylcholine diacyl C38:3
PC aa C38:4	Phosphatidylcholine diacyl C38:4
PC aa C38:5	Phosphatidylcholine diacyl C38:5
PC aa C38:6	Phosphatidylcholine diacyl C38:6
PC aa C40:1	Phosphatidylcholine diacyl C40:1
PC aa C40:2	Phosphatidylcholine diacyl C40:2
PC aa C40:3	Phosphatidylcholine diacyl C40:3
PC aa C40:4	Phosphatidylcholine diacyl C40:4
PC aa C40:5	Phosphatidylcholine diacyl C40:5
PC aa C40:6	Phosphatidylcholine diacyl C40:6
PC aa C42:0	Phosphatidylcholine diacyl C42:0
PC aa C42:1	Phosphatidylcholine diacyl C42:1
PC aa C42:2	Phosphatidylcholine diacyl C42:2
PC aa C42:4	Phosphatidylcholine diacyl C42:4
PC aa C42:5	Phosphatidylcholine diacyl C42:5
PC aa C42:6	Phosphatidylcholine diacyl C42:6
**ACYL-ALKYL PHOSPHATIDYLCHOLINES**	
PC ae C30:0	Phosphatidylcholine acyl-alkyl C30:0
PC ae C30:1	Phosphatidylcholine acyl-alkyl C30:1
PC ae C30:2	Phosphatidylcholine acyl-alkyl C30:2
PC ae C32:1	Phosphatidylcholine acyl-alkyl C32:1
PC ae C32:2	Phosphatidylcholine acyl-alkyl C32:2
PC ae C34:0	Phosphatidylcholine acyl-alkyl C34:0
PC ae C34:1	Phosphatidylcholine acyl-alkyl C34:1
PC ae C34:2	Phosphatidylcholine acyl-alkyl C34:2
PC ae C34:3	Phosphatidylcholine acyl-alkyl C34:3
PC ae C36:0	Phosphatidylcholine acyl-alkyl C36:0
PC ae C36:1	Phosphatidylcholine acyl-alkyl C36:1
PC ae C36:2	Phosphatidylcholine acyl-alkyl C36:2
PC ae C36:3	Phosphatidylcholine acyl-alkyl C36:3
PC ae C36:4	Phosphatidylcholine acyl-alkyl C36:4
PC ae C36:5	Phosphatidylcholine acyl-alkyl C36:5
PC ae C38:0	Phosphatidylcholine acyl-alkyl C38:0
PC ae C38:1	Phosphatidylcholine acyl-alkyl C38:1
PC ae C38:2	Phosphatidylcholine acyl-alkyl C38:2
PC ae C38:3	Phosphatidylcholine acyl-alkyl C38:3

PC ae C38:4	Phosphatidylcholine acyl-alkyl C38:4
PC ae C38:5	Phosphatidylcholine acyl-alkyl C38:5
PC ae C38:6	Phosphatidylcholine acyl-alkyl C38:6
PC ae C40:1	Phosphatidylcholine acyl-alkyl C40:1
PC ae C40:2	Phosphatidylcholine acyl-alkyl C40:2
PC ae C40:3	Phosphatidylcholine acyl-alkyl C40:3
PC ae C40:4	Phosphatidylcholine acyl-alkyl C40:4
PC ae C40:5	Phosphatidylcholine acyl-alkyl C40:5
PC ae C40:6	Phosphatidylcholine acyl-alkyl C40:6
PC ae C42:0	Phosphatidylcholine acyl-alkyl C42:0
PC ae C42:1	Phosphatidylcholine acyl-alkyl C42:1
PC ae C42:2	Phosphatidylcholine acyl-alkyl C42:2
PC ae C42:3	Phosphatidylcholine acyl-alkyl C42:3
PC ae C42:4	Phosphatidylcholine acyl-alkyl C42:4
PC ae C42:5	Phosphatidylcholine acyl-alkyl C42:5
PC ae C44:3	Phosphatidylcholine acyl-alkyl C44:3
PC ae C44:4	Phosphatidylcholine acyl-alkyl C44:4
PC ae C44:5	Phosphatidylcholine acyl-alkyl C44:5
PC ae C44:6	Phosphatidylcholine acyl-alkyl C44:6
**SPHINGOMYELINS**	
SM (OH) C14:1	Hydroxysphingomyelin C14:1
SM (OH) C16:1	Hydroxysphingomyelin C16:1
SM (OH) C22:1	Hydroxysphingomyelin C22:1
SM (OH) C22:2	Hydroxysphingomyelin C22:2
SM (OH) C24:1	Hydroxysphingomyelin C24:1
SM C16:0	Sphingomyelin C16:0
SM C16:1	Sphingomyelin C16:1
SM C18:0	Sphingomyelin C18:0
SM C18:1	Sphingomyelin C18:1
SM C20:2	Sphingomyelin C20:2
SM C22:3	Sphingomyelin C22:3
SM C24:0	Sphingomyelin C24:0
SM C24:1	Sphingomyelin C24:1
SM C26:0	Sphingomyelin C26:0
SM C26:1	Sphingomyelin C26:1
**SUGARS**	
H1	Hexose

Figure S1. Heatmap of the analyzed metabolites. Each row represents a subject and each column indicates a metabolite.

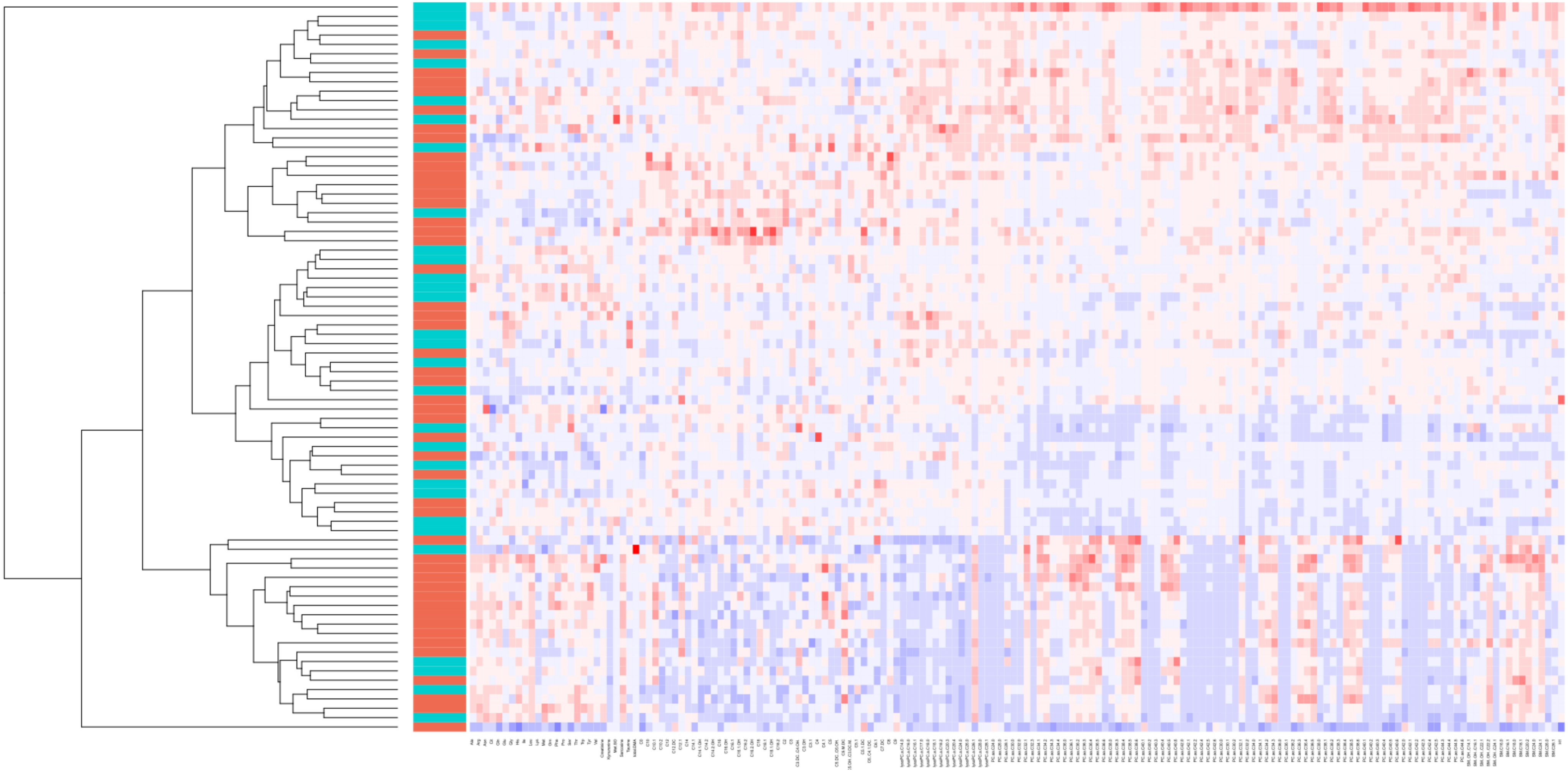

Figure S2. Correlation between metabolites.

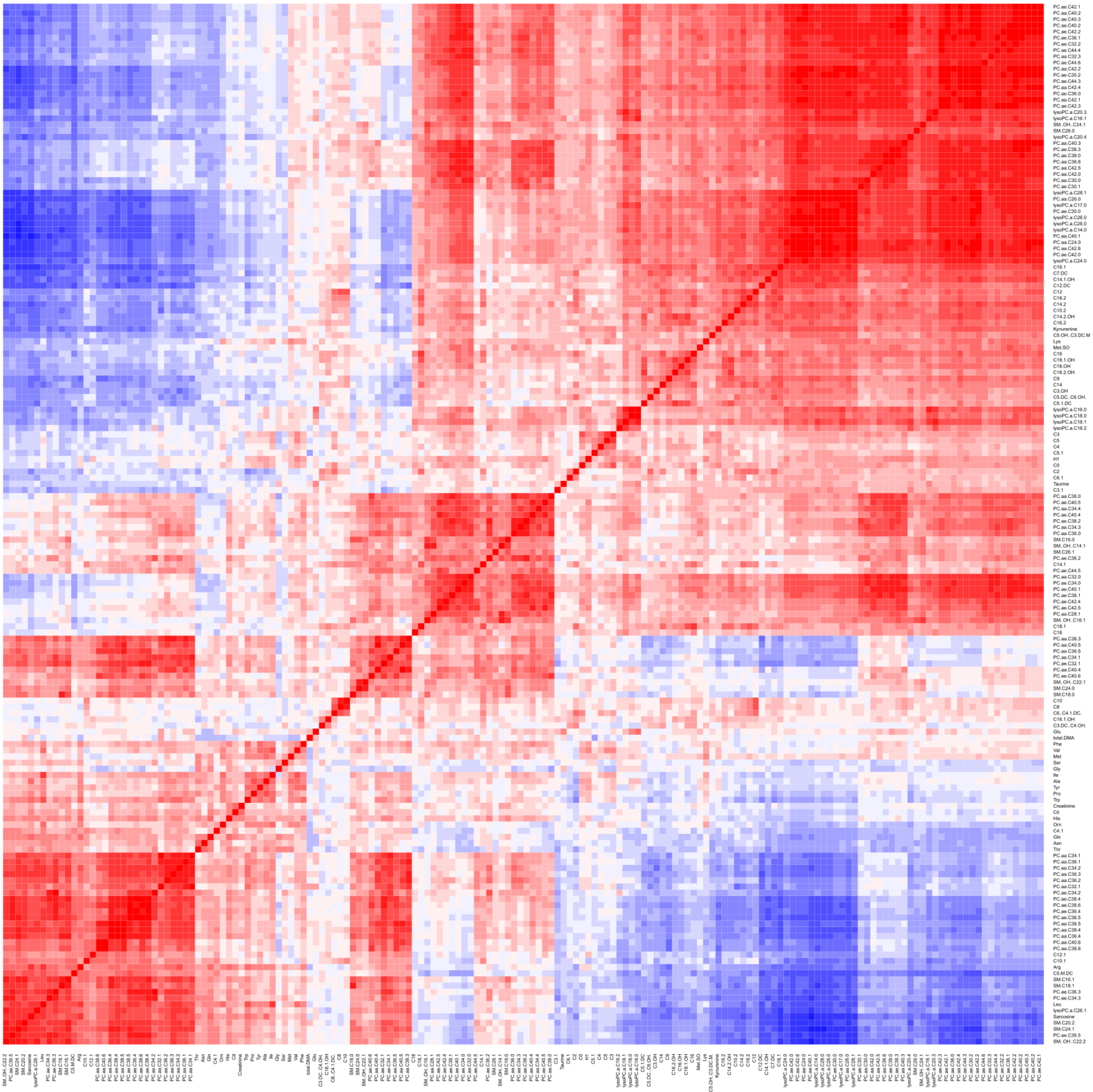

Figure S3. Percentage of variation for the first 9 principal components.

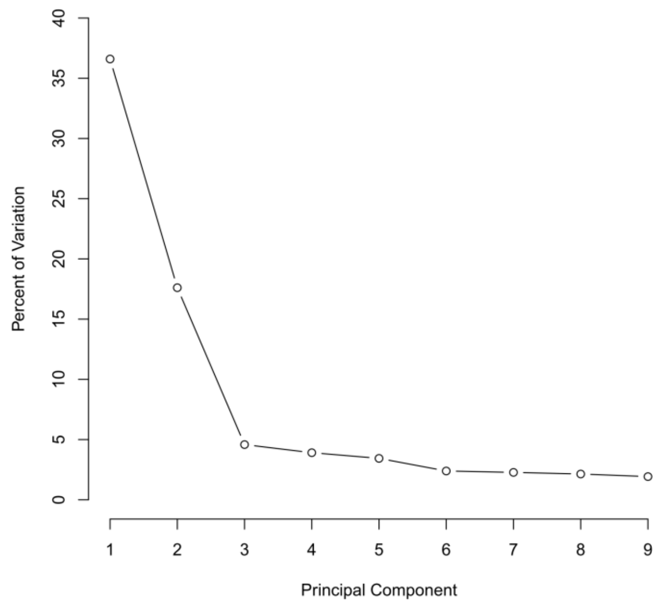

Figure S4. Score plot for the first 9 principal components.

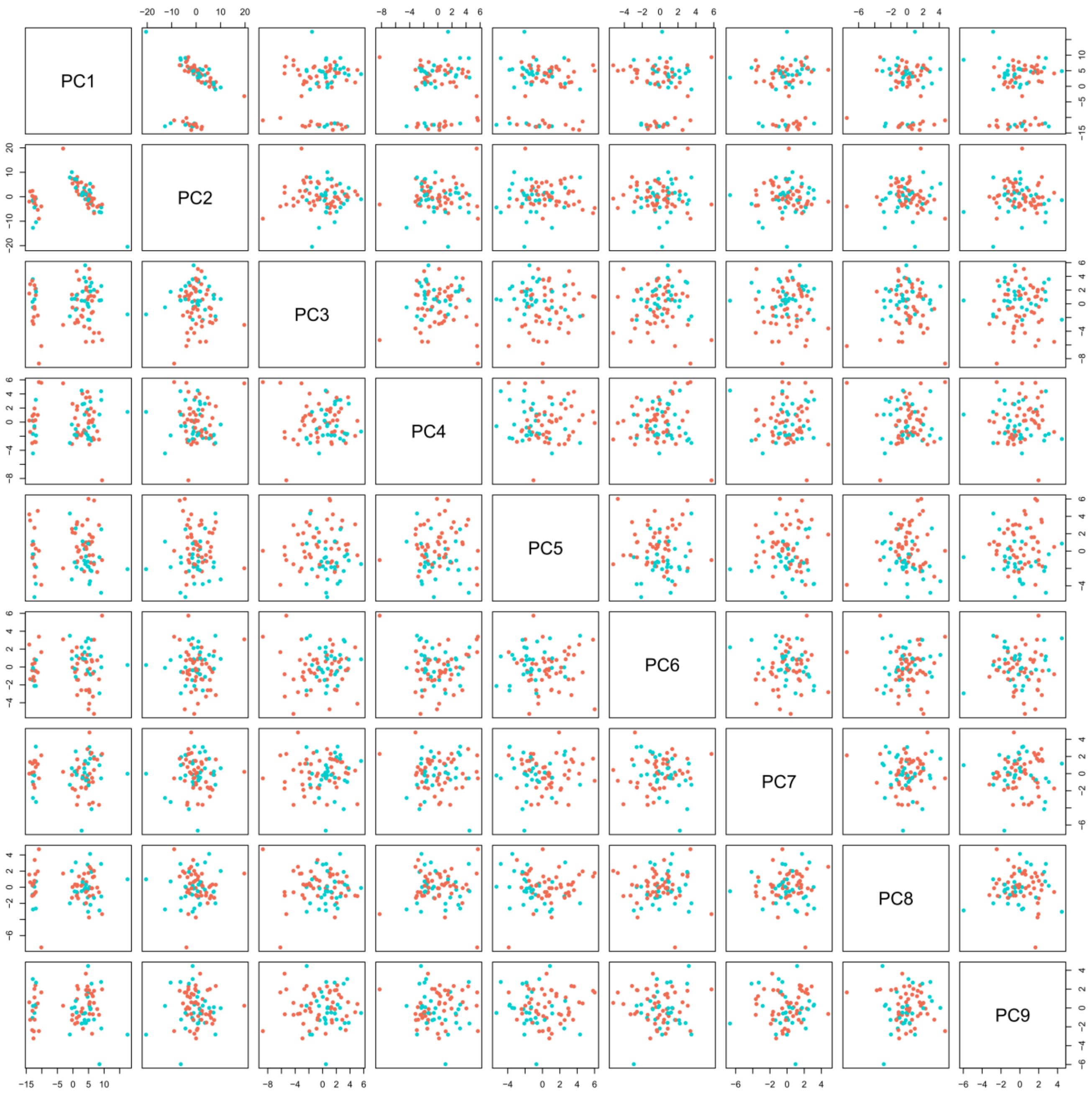

Supplement: Supplementary file 1 [file nutrients-09-00642-s001.zip › nutrients-197065-supplementary.pdf]
